# Protective Effects of Astragaloside IV against Amyloid Beta1-42 Neurotoxicity by Inhibiting the Mitochondrial Permeability Transition Pore Opening

**DOI:** 10.1371/journal.pone.0098866

**Published:** 2014-06-06

**Authors:** Qinru Sun, Ning Jia, Weixi Wang, Hui Jin, Jiehua Xu, Haitao Hu

**Affiliations:** Department of Human Anatomy and Histo-Embryology, Xi'an Jiaotong University Health Science Center, Xi'an, Shaanxi, China; Virginia Commonwealth University, United States of America

## Abstract

Mitochondrial dysfunction caused by amyloid β-peptide (Aβ) plays an important role in the pathogenesis of Alzheimer disease (AD). Substantial evidence has indicated that the mitochondrial permeability transition pore (mPTP) opening is involved in Aβ-induced neuronal death and reactive oxygen species (ROS) generation. Astragaloside IV (AS-IV), one of the major active constituents of *Astragalus membranaceus*, has been reported as an effective anti-oxidant for treating neurodegenerative diseases. However, the molecular mechanisms still need to be clarified. In this study, we investigated whether AS-IV could prevent Aβ1-42-induced neurotoxicity in SK-N-SH cells via inhibiting the mPTP opening. The results showed that pretreatment of AS-IV significantly increased the viability of neuronal cells, reduced apoptosis, decreased the generation of intracellular reactive oxygen species (ROS) and decreased mitochondrial superoxide in the presence of Aβ1-42. In addition, pretreatment of AS-IV inhibited the mPTP opening, rescued mitochondrial membrane potential (Δ*Ψ*m), enhanced ATP generation, improved the activity of cytochrome c oxidase and blocked cytochrome c release from mitochondria in Aβ1-42 rich milieu. Moreover, pretreatment of AS-IV reduced the expression of Bax and cleaved caspase-3 and increased the expression of Bcl-2 in an Aβ1-42 rich environment. These data indicate that AS-IV prevents Aβ1-42-induced SK-N-SH cell apoptosis via inhibiting the mPTP opening and ROS generation. These results provide novel insights of AS-IV for the prevention and treatment of neurodegenerative disorders such as AD.

## Introduction

Alzheimer's disease (AD) is the most common neurodegenerative disorder in the elderly resulting in neuronal loss and impaired cognitive. The pathological hallmarks of AD include neurofibrillary tangles and massive accumulated amyloid beta (Aβ) in the brain [1]. A lot of evidence *in vitro* and *in vivo* indicate that oligomer Aβ1-42 exerts neurotoxicity including intracellular calcium perturbation, reactive oxygen species (ROS) accumulation and pro-apoptosis factor activation [2]–[5]. Mitochondria play important roles in accommodating cellular redox state and maintaining intracellular calcium homeostasis. Studies have shown that Aβ1-42 could cause mitochondrial dysfunctions such as deficiency of glucose metabolism, deactivation of key enzymes for oxidative phosphorylation and accumulation of mitochondrial reactive free radicals [6], [7]. These studies suggest that Aβ1-42 is linked to mitochondrial dysfunction in cortical neurons of AD patients and AD mouse models.

The mitochondrial permeability transition pore (mPTP) has a central role in neuronal cell death in neurodegenerative disease. The mPTP is thought to consist of the voltage-dependent anion channel (VDAC) in the outer mitochondrial membrane, the adenine nucleotide translocator (ANT) in the inner mitochondrial membrane and cyclophilin D (CypD) in the mitochondrial matrix. Many factors such as high concentration of Ca^2+^ and ROS appear to induce the mPTP opening [8]. The opening of the mPTP results in mitochondrial depolarization and mitochondrial membrane potential (Δ*Ψ*m) dissipation followed by progressive mitochondrial swelling and the loss of soluble components of the respiratory chain, which eventually leads to rupture of the outer mitochondrial membrane and leakage of proteins from mitochondria to cytosol [9]. A large body of evidence shows that the mPTP opening is involved in the pathogenesis of AD. It is believed that several mitochondrial proteins interact with Aβ, which results in the opening of the mPTP. Aβ-induced mPTP opening leads to Δ*Ψ*m collapse and pro-apoptotic factor release from mitochondria to cytosol [10]–[12].

There is an important theory which states that the members of the Bcl-2 family such as Bcl-2 and Bax exert their pro- or anti-apoptotic effect through regulating the opening of the mPTP [13]. Recently, experimental evidence indicates that Bax is required for mPTP-dependent cell death [14]. So far, studies concerning the relationship between Bcl-2 and the mPTP opening have been rarely reported. Furthermore, many investigations show that the expression of Bax is increased and the expression of Bcl-2 is decreased in Aβ1-42-induced neuronal apoptosis [15]–[17]. These results imply that Aβ1-42 induces the mPTP opening which might be regulated by Bcl-2 family proteins. In addition, in AD patients and AD mouse models, accumulation of intracellular ROS triggers the mPTP opening which finally leads to mitochondrial dysfunction and cell apoptosis [18]–[20].

**Figure 1:**
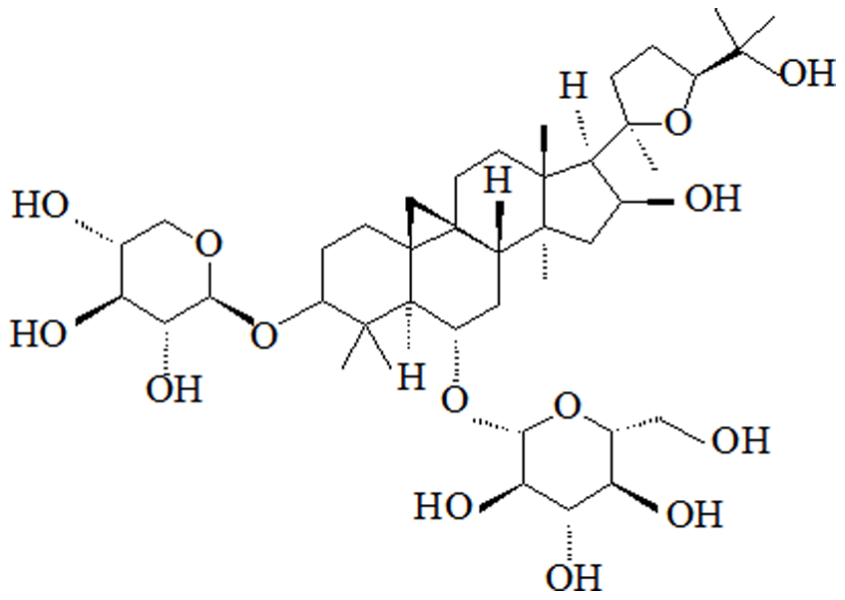
Chemical structure of astragaloside IV.

Astragaloside IV (AS-IV, chemical structure shown in Fig. 1), is a small molecular (MW = 784 Da) saponin purified from *Astragalus membranaceus*, that has been routinely used in China to treat chronic diseases [21]. It has been reported that AS-IV has an antioxidant effect and the accepted underlying mechanisms include modulation of energy metabolism and Ca^2+^ homeostasis [22]–[25]. Moreover, AS-IV shows neuroprotective effects on promoting axonal regeneration and reconstruction of neuronal synapses [26]. However, the protective effects of AS-IV against Aβ1-42-induced mitochondrial dysfunction and neuronal death still need to be elucidated. In the present study, we investigated the effects of AS-IV against Aβ1-42-induced mPTP opening in SK-N-SH cells and elucidated the underlying molecular mechanisms.

## Materials and Methods

### Cell Lines, Culture Conditions, and Treatment with Reagents

Human SK-N-SH neuroblastoma cells (ATCC) were cultured in DMEM supplemented with 10% (v/v) heat-inactivated fetal calf serum (Invitrogen) and 100 U/ml penicillin/streptomycin (Invitrogen). Cells were maintained at 37°C in humidified 5% CO_2_ and 95% air and the culture medium was replaced every 2 days. All experiments were carried out 24–48 h after cells were seeded in culture plates. Cells were permitted to attach for 24 h and grown to 75% confluence. AS-IV (Soboo Biotech, purity >99%) was dissolved in DMSO to the concentration of 10 mg/ml as a stock solution. Oligomer Aβ1-42 (Sigma) was prepared as described [27]. The highest final concentration of DMSO in the medium was 0.1% to avoid affecting cell viability. All treated SK-N-SH cells were divided into 3 groups as follows: vehicle group (cells treated with 0.1% DMSO), Aβ1-42 group (cells treated with 5 µM Aβ1-42) and AS-IV+ Aβ1-42 group (cells pretreated with AS-IV at various concentration (10, 25, 50 µM) 2 h prior to 5 µM Aβ1-42). All experiments were carried out after incubation for 24 h.

### Cell viability assay

Cell viability was measured in a 96-well plate using a quantitative colorimetric assay with 3-(4, 5-dimethylthiazol-2-yl)-2, 5-diphenyltetrazolium bromide (MTT). Briefly, SK-N-SH cells were cultured on 96-well plates. After treatments above, the MTT solution (5 mg/mL) was added to each well (20 µL/well) at a final concentration of 0.5 mg/mL and incubated at 37°C for 4 h. The MTT solution was removed gently and 200 µL of DMSO was added to each well for 15 min incubation. The absorbance of each sample was measured at 490 nm using a microplate reader (Bio-Rad).

### Measurement of cellular ROS and mitochondrial superoxide

Generation of intracellular reactive oxygen species (ROS) was monitored by using the 2′,7′-Dichlorofluorescin diacetate (DCFH-DA) fluorescent probe (Invitrogen) and mitochondrial superoxide levels were monitored by using the fluorescent probe MitoSOX Red (Invitrogen). Briefly, after treatments, SK-N-SH cells were incubated with 10 µM DCFH-DA or 5 µM MitoSOX Red at 37°C for 30 min. After washing twice with PBS, the fluorescence intensity was observed by using OLYMPUS FV1000 confocal microscopy. The intensity of fluorescence staining was analyzed with Image J software (NIH). Mitochondria were counterstained with Mitotracker Red (red fluorescence) or Mitotracker Green (green fluorescence) (Invitrogen), respectively.

### Mitochondrial permeability transition pore (mPTP) Assay

The opening of the mPTP in cultured SK-N-SH cells was assessed by the Calcein/Co2+-quenching technique as described by using the MitoProbe Transition Pore Assay Kit (Molecular Probes) according to the manufacturer's instructions. Briefly, after treatments, SK-N-SH cells were loaded with 1 µM Calcein-AM (green), 2 mM CoCl2 and 20 nM MitoTracker Red (Invitrogen) at 37°C for 20 min in phenol red free Hank's buffered salt solution (Invitrogen). After washings, live cells were imaged using OLYMPUS FV1000 confocal microscopy with appropriate excitation and emission filters for fluorescein. The mPTP inhibitor cyclosporin A (CsA) (1 µM) (LC laboratories) was applied as a positive control.

### Analysis of Apoptotic Parameters

Terminal deoxynucleotidyl transferase dUTP nick end labeling (TUNEL) assays were conducted by In Situ Cell Death Detection Kit AP (Roche) according to the manufacturer's instruction. Briefly, after treatments, SK-N-SH cells were fixed in 4% paraformaldehyde and stained with 10 mg/mL 4',6'-diamidino-2-phenylindole hydrochloride (DAPI) (Invitrogen) at 37°C for 10 min. Stained cells were observed under a fluorescence microscope (Olympus). The TUNEL-positive cells were counted and the ratio of apoptotic cells to total cells (DAPI-stained) was determined.

### ATP level measurement

The ATP level was measured by the ATP Determination Kit (Roche) according to the manufacturer's instruction. After treatments, SK-N-SH cells were homogenized by using cell lysis buffer, incubated on ice for 15 min, and centrifuged at 14,000 x *g* for 15 min at 4°C. Subsequent supernatants were collected and chemiluminescence was measured by using a Beckman Coulter DTX880 (Beckman) with an integration time of 10 seconds.

### Measurement of mitochondrial membrane potential (ΔΨm)

Δ*Ψ*m was detected by TMRM staining according to manufacturer's instructions. After treatments, SK-N-SH cells were stained by TMRM (200 nM, Invitrogen) for 30 min at 37°C and then washed with medium twice. The cells were observed and imaged with OLYMPUS FV1000 confocal microscopy. The intensity of TMRM was analyzed with Image J software (NIH). Mitochondria were counterstained with Mitotracker Green (Invitrogen).

### Detection of the activity of cytochrome c oxidase (CcO activity)

Cytochrome c oxidase activity was measured by using the cytochrome c oxidase kit (Sigma). After treatments, SK-N-SH cells were collected. A total volume of 100 µL mixture of cell lysis buffer and enzyme solution was added into 950 µL assay buffer. The reaction was initiated by adding 50 µL ferrocytochrome c substrate solution. The fluorescence of the final mixture at 550 nm was recorded with a SmartSpec Plus spectrophotometer (Bio-rad).

### Cytochrome c release assay

Cytochrome c release was measured by using the Cytochrome c Releasing Apoptosis Assay Kit (Abcam). Briefly, after treatments, cells were collected by centrifugation at 600 x *g* for 5 min at 4°C and washed for twice. Cells were homogenized and isolated as cytosolic and mitochondrial extraction by employing the reagents. 10 µg each of the cytosolic and mitochondrial fraction was loaded on a 12% SDS-PAGE. A standard Western blot procedure was done and probed with monoclonal mouse anti-cytochrome c antibody (Cell signaling). Cytochrome c oxidase subunit IV (COX IV, Cell signaling) and polyclonal mouse anti β-actin (Sigma) were used as loading controls.

### Protein extraction and Western blot analysis

After treatments, cells were washed twice with ice-cold PBS, and then cells were homogenized at 1∶5 (wt/vol) in an ice-cold lysis buffer. Samples were resolved by SDS-PAGE and transferred to Hybond-ECL nitrocellulose membranes (Bio-rad). The blots were probed with the following primary antibodies: polyclonal mouse anti β-actin (Sigma), monoclonal mouse anti-Bax (Cell Signaling), monoclonal mouse anti-Bcl-2 (Cell Signaling) and polyclonal rabbit anti-cleaved caspase-3 (Santa Cruz) followed by incubation with species-matched horseradish peroxidase-conjugated secondary antibodies. The blots were developed with a chemiluminescence substrate solution (Pierce) and exposed to X-ray film. The optical density of immunoreactive bands was quantified using Bio-rad software.

### Statistical analysis

All experiments were repeated more than three times. All values were expressed as mean ± standard error (SE). Statistical significance was determined via one-way analysis of variance (ANOVA) followed by the Tukey-Kramer test for multiple comparisons when appropriate using SPSS software (version 16.0, SPSS). A value of *P*<0.05 was considered to be statistically significant.

## Results

### Pretreatment of AS-IV prevented Aβ1-42-induced neuronal cell death in SK-N-SH cells

**Figure 2:**
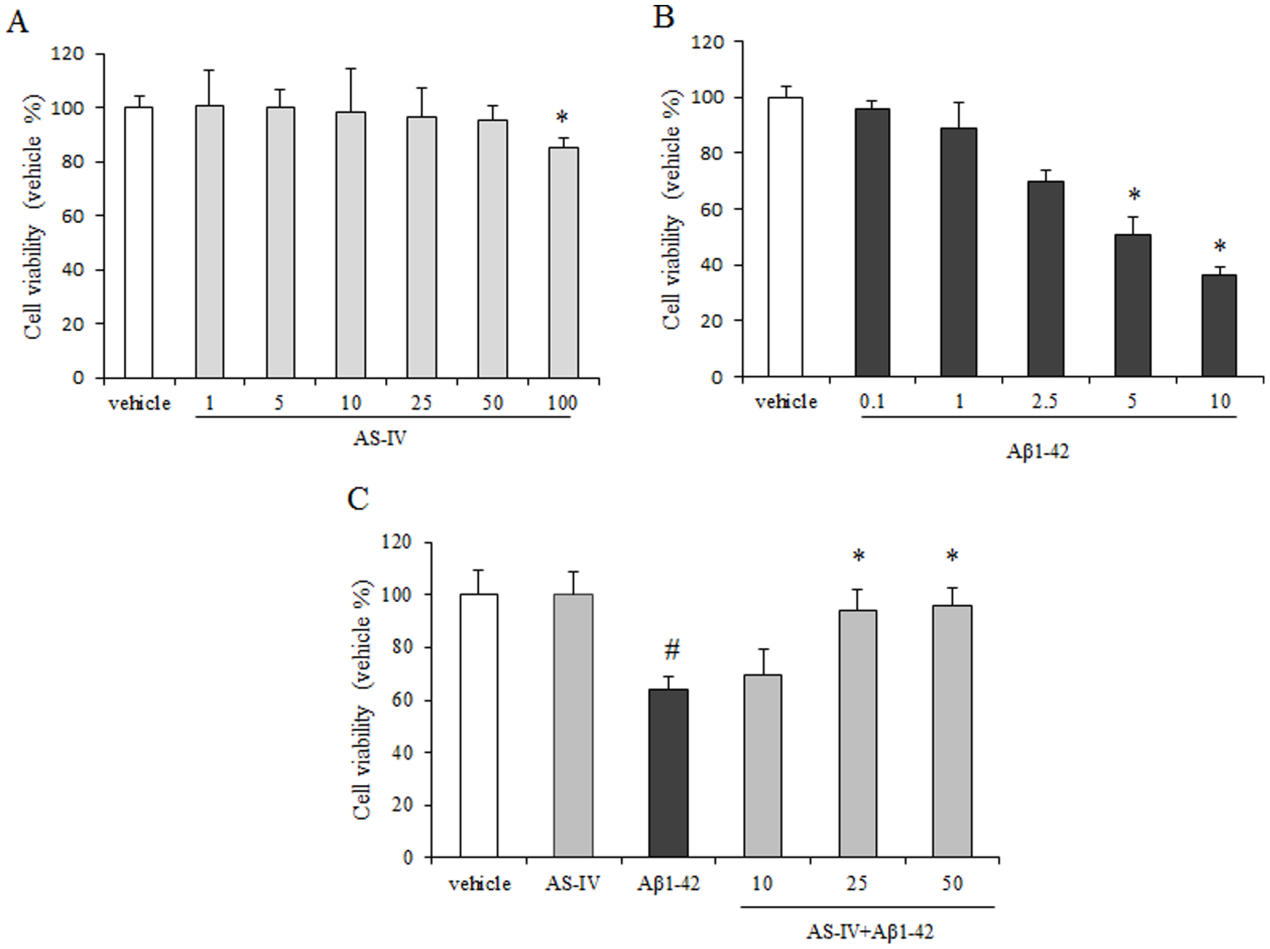
AS-IV pretreatment attenuates Aβ1-42-induced SK-N-SH cell death. A. SK-N-SH cells were treated with different concentrations (0–100 µM) of AS-IV for 24 h. B. SK-N-SH cells were treated with different concentrations (0–10 µM) of Aβ1-42 for 24 h. C. SK-N-SH cells were pretreated with different concentrations of AS-IV (10, 25, 50 µM) for 2 h and then incubated with Aβ1-42 (5 µM) for 24 h. Viability of cells was detected by MTT assay. Percentage of cell viability was relative to the untreated vehicle cells. ^#^ *P*<0.01 *vs* vehicle; ^*^ *P*<0.01 *vs* Aβ1-42 (n = 3).

To test the effect of AS-IV, SK-N-SH cells were subjected to various concentrations of AS-IV for 24 h, and no significant difference was observed in cell viability assessed by the MTT assay among the AS-IV (1, 5, 10, 25, 50 µM) group and the vehicle group. However, cells treated with higher dose of AS-IV (100 µM) showed about 10% reduction of cell viability (Fig. 2A, *P*<0.01). Concentrations of 10, 25, 50 µM of AS-IV were selected to subsequent experiments. To examine the toxicity for oligomer Aβ1-42, SK-N-SH cells were treated with oligomer Aβ1-42 (0.1, 1, 2.5, 5, 10 µM) for 24 h and displayed a dose-dependent reduction in cell viability. Lower concentration of Aβ1-42 (1, 2.5 µM) slightly damaged the cells, and cells were severely impaired by 10 µM Aβ1-42. Application of 5 µM oligomer Aβ1-42 showed a nearly 50% reduction in cell viability and 5 µM Aβ1-42 was selected to be used in the subsequent experiments (Fig. 2B).

AS-IV at 10, 25 and 50 µM was added to SK-N-SH cells 2 h prior to the addition of 5 µM Aβ1-42. Pretreatment of 25 and 50 µM AS-IV significantly increased cell viability in a dose-dependent manner. The cell viability in AS-IV pretreatment group was still lower than those in the vehicle group (Fig. 2C, *P*<0.01). Pretreatment of AS-IV at 10 µM did not show a significant difference compared with Aβ1-42 treatment.

### AS-IV attenuated Aβ1-42 induced mitochondrial dysfunction

**Figure 3:**
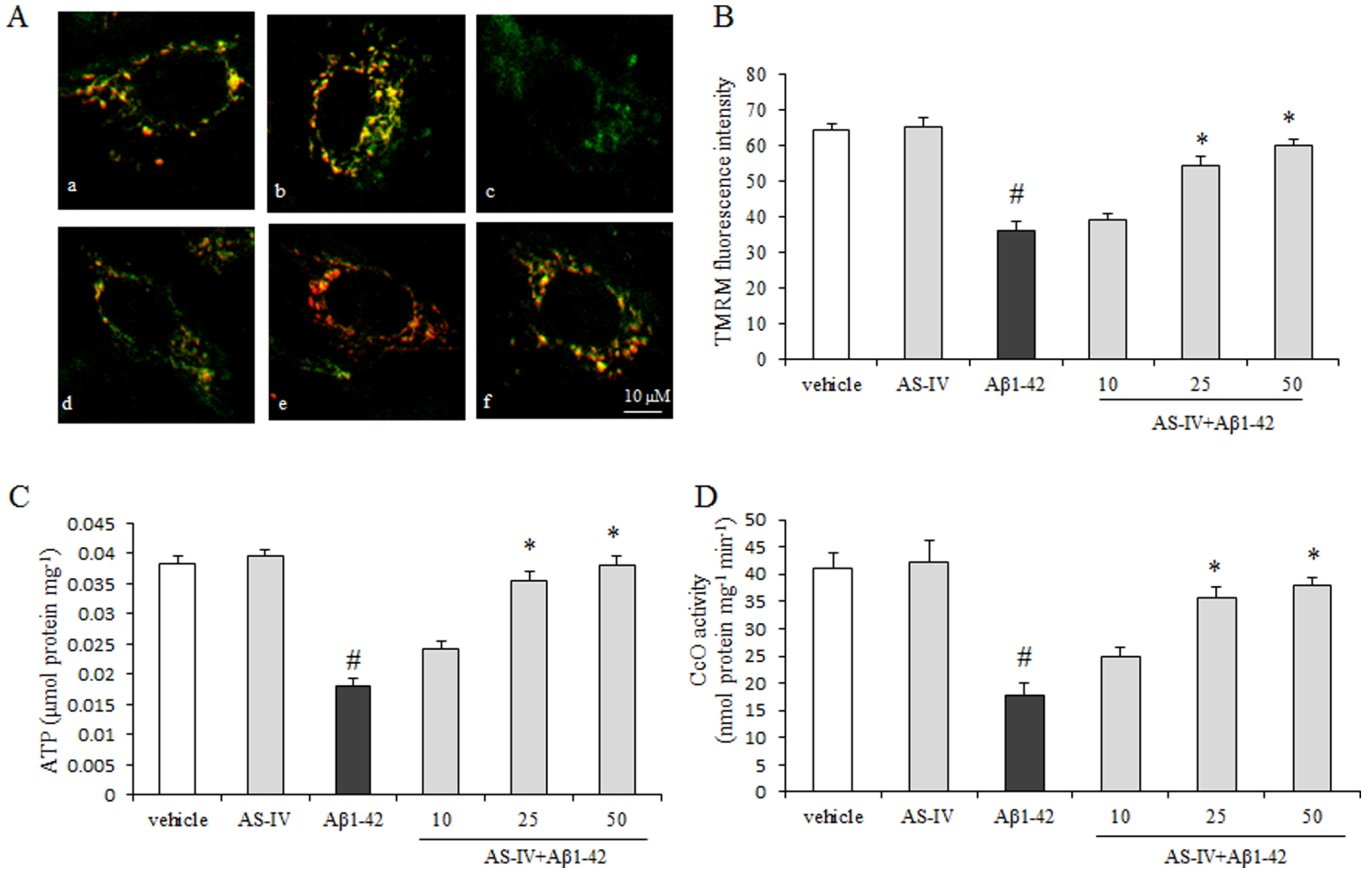
AS-IV attenuated Aβ1-42-induced mitochondrial dysfunction. A. A representative image of mitochondrial membrane potential detection by TMRM staining (red fluorescence). Mitochondria were counterstained with Mitotracker Green (green fluorescence). (a) vehicle; (b) 50 µM AS-IV; (c) 5 µM Aβ1-42; (d, e, f) 10, 25, 50 µM AS-IV+ 5 µM Aβ1-42, respectively. B. Quantification analysis of Δ*Ψ*m. ^#^ *P*<0.01 *vs* vehicle; ^*^ *P*<0.01 *vs* Aβ1-42 (n = 6). C. ATP detection. D. Cytochrome c oxidase activity detection. Aβ1-42 significantly decreased both CcO activity and ATP which was reversed by pretreatment with AS-IV (25, 50 µM). ^#^ *P*<0.01 *vs* vehicle; ^*^ *P*<0.01 *vs* Aβ1-42 (n = 6). Scale bar = 10 µm.

To explore the potential role of AS-IV in Aβ1-42-induced neuronal cell death, we examined the mitochondrial function by testing mitochondrial membrane potential (Δ*Ψ*m), ATP level and cytochrome c oxidase (CcO) in SK-N-SH cells. Firstly, we employed TMRM as an indicator of mitochondrial membrane potential (Δ*Ψ*m). Aβ1-42 treated SK-N-SH cells showed a significant decrease in red fluorescence intensity compared with cells from the vehicle group (*P*<0.01). Cells pretreated with 25 or 50 µM AS-IV at showed higher red fluorescence intensity compared with cells treated with Aβ1-42 alone for 24h (*P*<0.01). There was no significant difference between the 10 µM AS-IV pretreatment group and the Aβ1-42 group (*P*<0.01) (Fig. 3A, B). Secondly, we measured ATP level. As shown in Fig. 3C, ATP generation in the Aβ1-42 group was decreased compared with the vehicle group. In the presence of AS-IV at 25 or 50 µM, ATP level was significantly increased compared with that in the Aβ1-42 group (*P*<0.01). Application of 10 µM AS-IV did not recover ATP level (*P*>0.05). Thirdly, the CcO activity was examined. Cells treated with 5 µM Aβ1-42 demonstrated significantly decreased CcO activity compared with that in the vehicle group (*P*<0.01). Notably, cells in the presence of AS-IV exhibited significantly increased CcO activity in a dose-dependent manner compared with cells treated with Aβ1-42 alone for 24 h (*P*<0.01). Pretreatment of 25 and 50 µM AS-IV significantly increased CcO activity in a dose-dependent manner. AS-IV at 10 µM did not show significant difference compared with that in the Aβ1-42 group (*P*>0.05) (Fig. 3D). Statistical analysis showed that the mitochondrial function of cells pretreated with AS-IV did not recovery fully to the level of the vehicle group. In the experiments to detect Δ*Ψ*m, ATP level or CcO activity, 50 µM AS-IV alone treatment did not show an insult to SK-N-SH cells.

### AS-IV inhibited Aβ1-42-induced cytochrome c release from mitochondria

**Figure 4:**
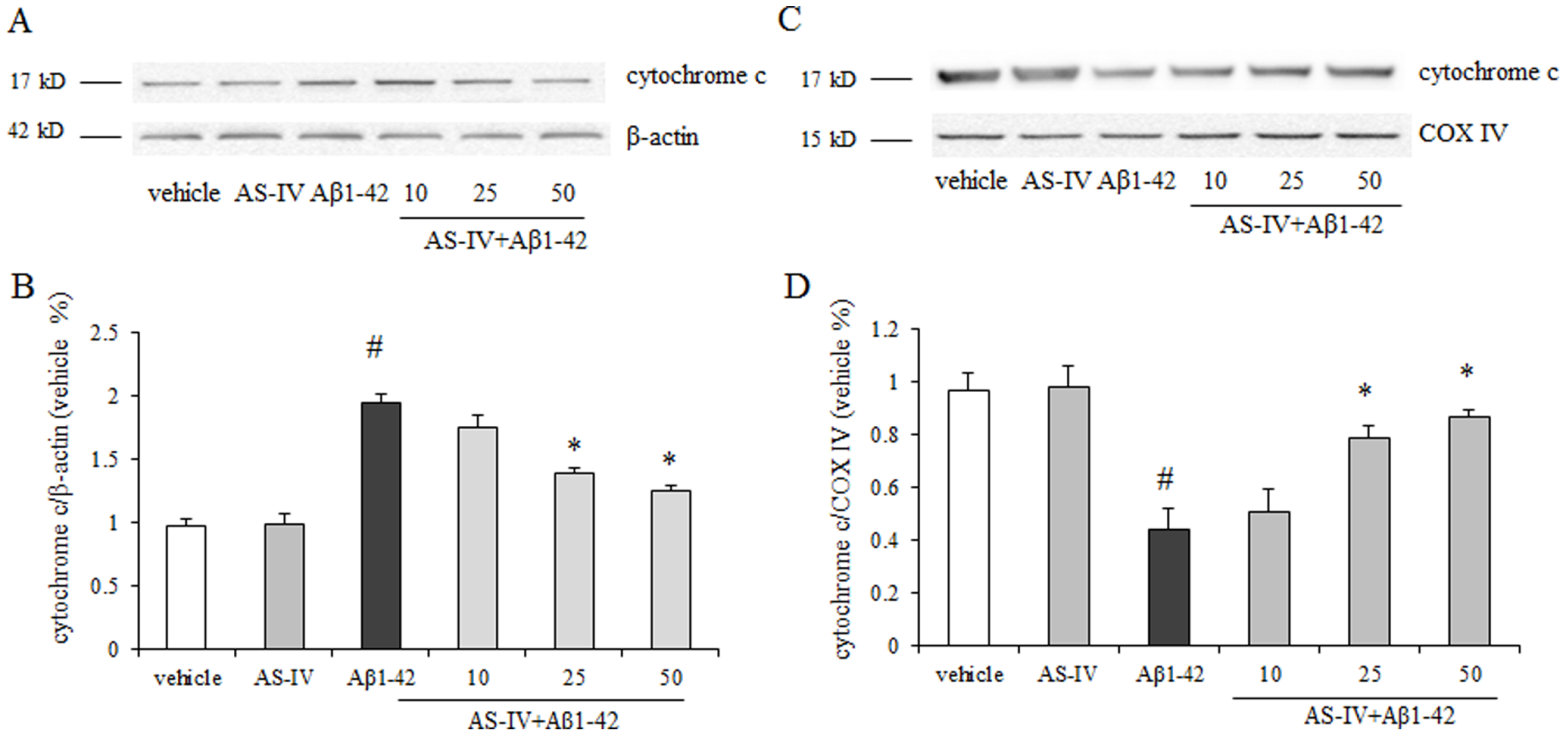
AS-IV inhibited Aβ1-42-induced cytochrome c release from mitochondria in SK-N-SH cells. A. A representative blots of immunoreactive bands for cytochrome c in cytosol. B. Data were expressed as fold-increase of cytochrome c relative to vehicle. Protein expression levels were normalized to β-actin. C. A representative blots of immunoreactive bands for cytochrome c in mitochondria. D. Data were expressed as fold-increase of cytochrome c relative to vehicle. Protein expression levels were normalized to COX IV. ^#^ *P*<0.01 *vs* vehicle; ^*^ *P*<0.01 *vs* Aβ1-42 (n = 4).

Release of cytochrome c from mitochondria to cytosol is considered as a key initial step in the mitochondria-mediated apoptotic process. We examined the levels of cytochrome c in cytosol and mitochondria by Western blot. Compared with the vehicle group, the Aβ1-42 group showed significantly higher levels of cytochrome c in cytosol (*P*<0.01). The levels of cytochrome c was significantly decreased with a dose-dependent manner in the presence of AS-IV (25, 50 µM) compared with that in the Aβ1-42 group (*P*<0.01) (Fig. 4A, B). Cells treated with 10 µM AS-IV did not show a significant decreasedid not. The results showed that compared with the vehicle group, the Aβ1-42 group showed significantly lower levels of cytochrome c in mitochondria (*P*<0.01). Cells pretreated with AS-IV at 25, 50 µM showed higher levels of cytochrome c in mitochondria than cells treated with Aβ1-42. Cells treated with 10 µM AS-IV did not show difference (Fig. 4C, D). 50 µM AS-IV alone treatment did not show an insult to SK-N-SH cells.

### Pretreatment of AS-IV inhibited Aβ1-42-induced apoptosis in SK-N-SH cells

**Figure 5:**
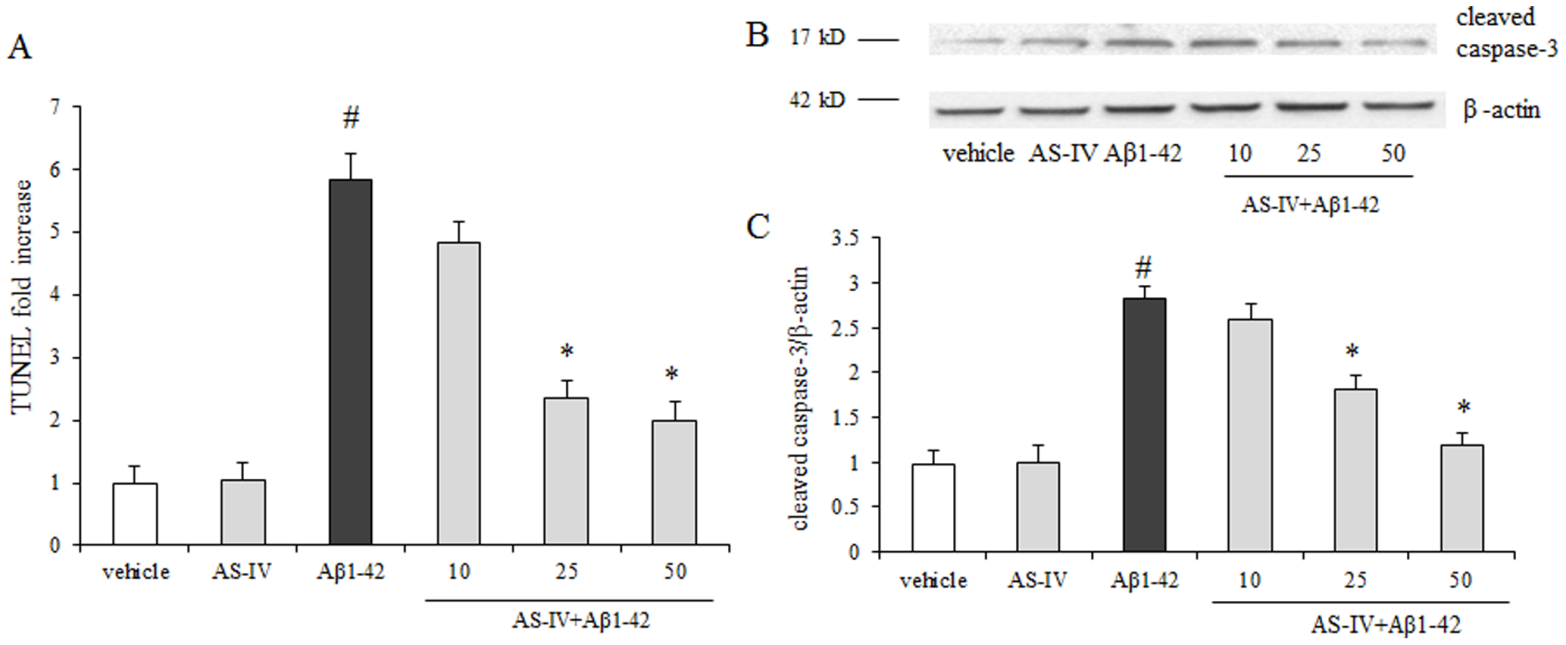
Protective effects of AS-IV on Aβ1-42-induced apoptosis in SK-N-SH cells. A. Detection of apoptosis by TUNEL assay in different groups. Percentage of TUNEL positive cells was relative to the untreated vehicle cells. ^#^ *P*<0.01 *vs* vehicle; ^*^ *P*<0.01 *vs* Aβ1-42 (n = 4). B. AS-IV inhibited Aβ1-42-induced activation of caspase-3 in SK-N-SH cells. A representative blots of immunoreactive bands for cleaved caspase-3 in SK-N-SH cells. C. Data were expressed as fold-increase of cleaved caspase-3 relative to vehicle. Protein expression levels were normalized to β-actin. ^#^ *P*<0.01 *vs* vehicle; ^*^ *P*<0.01 *vs* Aβ1-42 (n = 4).

Next, we investigated the protective effect of AS-IV against Aβ1-42-induced apoptosis in SK-N-SH cells. The Aβ1-42 group showed more TUNEL-positive cells compared with the vehicle group. Pretreatment of AS-IV at 25 or 50 µM significantly decreased TUNEL-positive cells numbers in a dose-dependent manner compared with Aβ1-42 treatment (*P*<0.01), though the apoptotic cells numbers were slightly more than that in the vehicle group. Few apoptotic cells were visible in the vehicle group. Pretreatment of 10 µM AS-IV did not show a significant difference compared with Aβ1-42 treatment (*P*>0.05) (Fig. 5A). To confirm the anti-apoptotic effect of AS-IV in the presence of Aβ1-42, we measured the expression of cleaved caspase-3 protein. As shown in Fig. 5B + Chttp://link.springer.com/article/10.1007/s11010-011-1219-1/fulltext.html - Fig3, cells in the Aβ1-42 group exhibited significantly increased level of cleaved caspase-3 protein compared with cells in the vehicle group (*P*<0.01). Pretreatment of AS-IV at 25 or 50 µM significantly decreased cleaved caspase-3 protein expression in a dose-dependent manner compared with the Aβ1-42 treatment, but the cleaved caspase-3 level is higher than that in the vehicle group (*P*<0.01). Cells pretreated with 10 µM AS-IV did not show significantly changes compared with cells treated with Aβ1-42 alone (*P*<0.01) (Fig. 5C). 50 µM AS-IV alone treatment did not show an insult to SK-N-SH cells.

### AS-IV blocked Aβ1-42-induced mPTP opening

**Figure 6:**
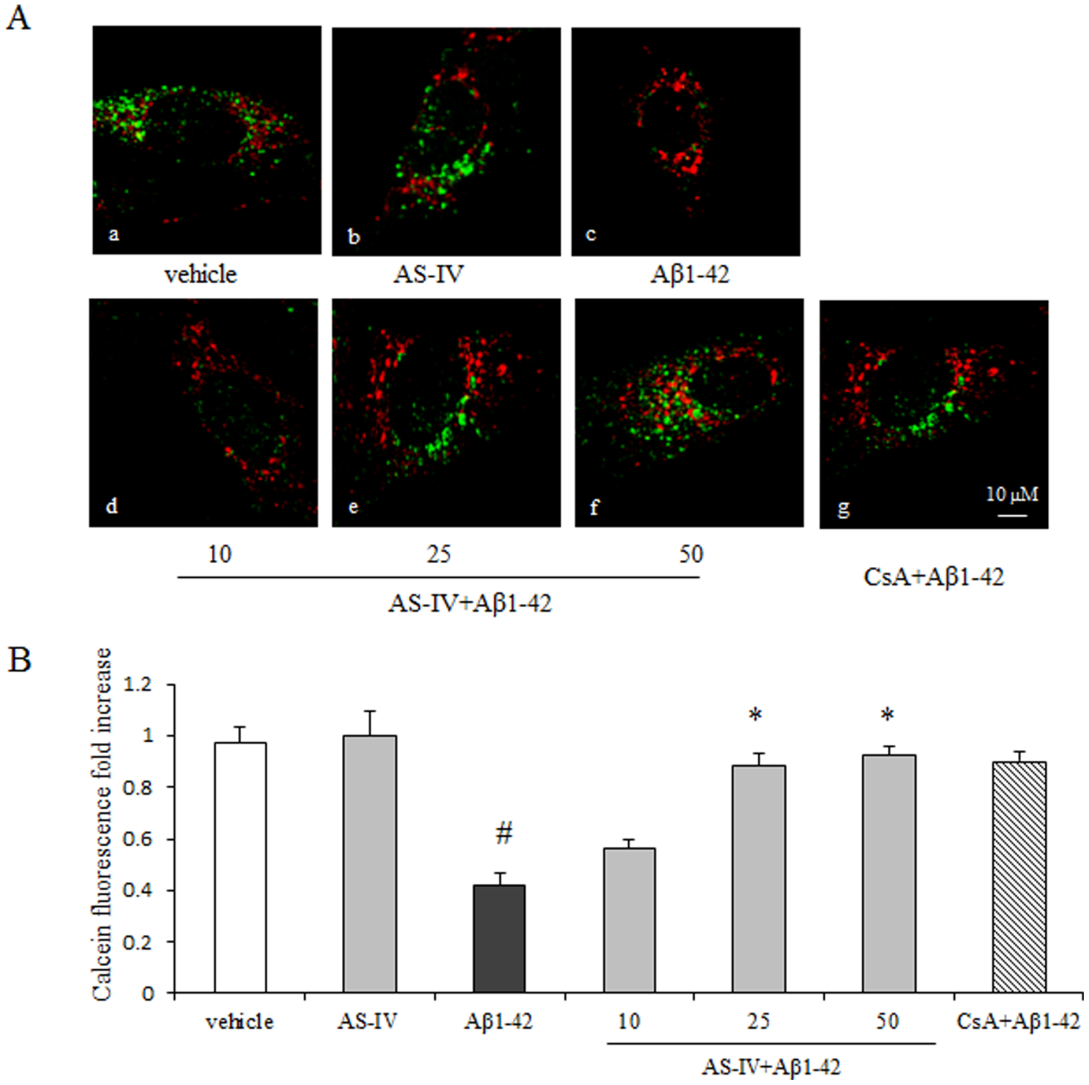
AS-IV blocked Aβ1-42-induced mPTP opening. A. Mitochondrial permeability transition pore detection by Calcein-AM staining. Mitochondria were counterstained with Mitotracker Red (red fluorescence). (a) vehicle; (b) 50 µM AS-IV; (c) 5 µM Aβ1-42; (c, d, e, f) 10, 25, 50 µM AS-IV+ 5 µM Aβ1-42; (g) 1 µM CsA+ 5 µM Aβ1-42, respectively. B. Quantification analysis of Calcein-AM staining relative to the vehicle group. 1 µM CsA was applied as positive control. ^#^ *P*<0.01 *vs* vehicle; ^*^ *P*<0.01 *vs* Aβ1-42 (n = 6). Scale bar = 10 µm

To investigate whether the mPTP opening was involved in AS-IV attenuating Aβ1-42-induced mitochondrial dysfunction, we employed Calcein-AM/cobalt chloride quenching method. Compared with the vehicle group, cells in the Aβ1-42 group showed a significantly lower level of green fluorescence (*P*<0.01). Pretreatment of AS-IV at 25 or 50 µM significantly increased Calcein fluorescence intensity compared with Aβ1-42 treatment alone but the intensity did not go back to the level of the vehicle group (*P*<0.01). 10 µM AS-IV pretreatment did not show a significant difference compared with Aβ1-42 treatment alone (*P*>0.05). The mPTP inhibitor CsA increased Calcein fluorescence intensity, confirming that Calcein quenching reflected the opening of the mPTP. 50 µM AS-IV alone treatment did not show an insult to SK-N-SH cells. (Fig. 6A, B).

### Pretreatment with AS-IV reduced Aβ1-42-induced ROS generation in SK-N-SH cells

**Figure 7:**
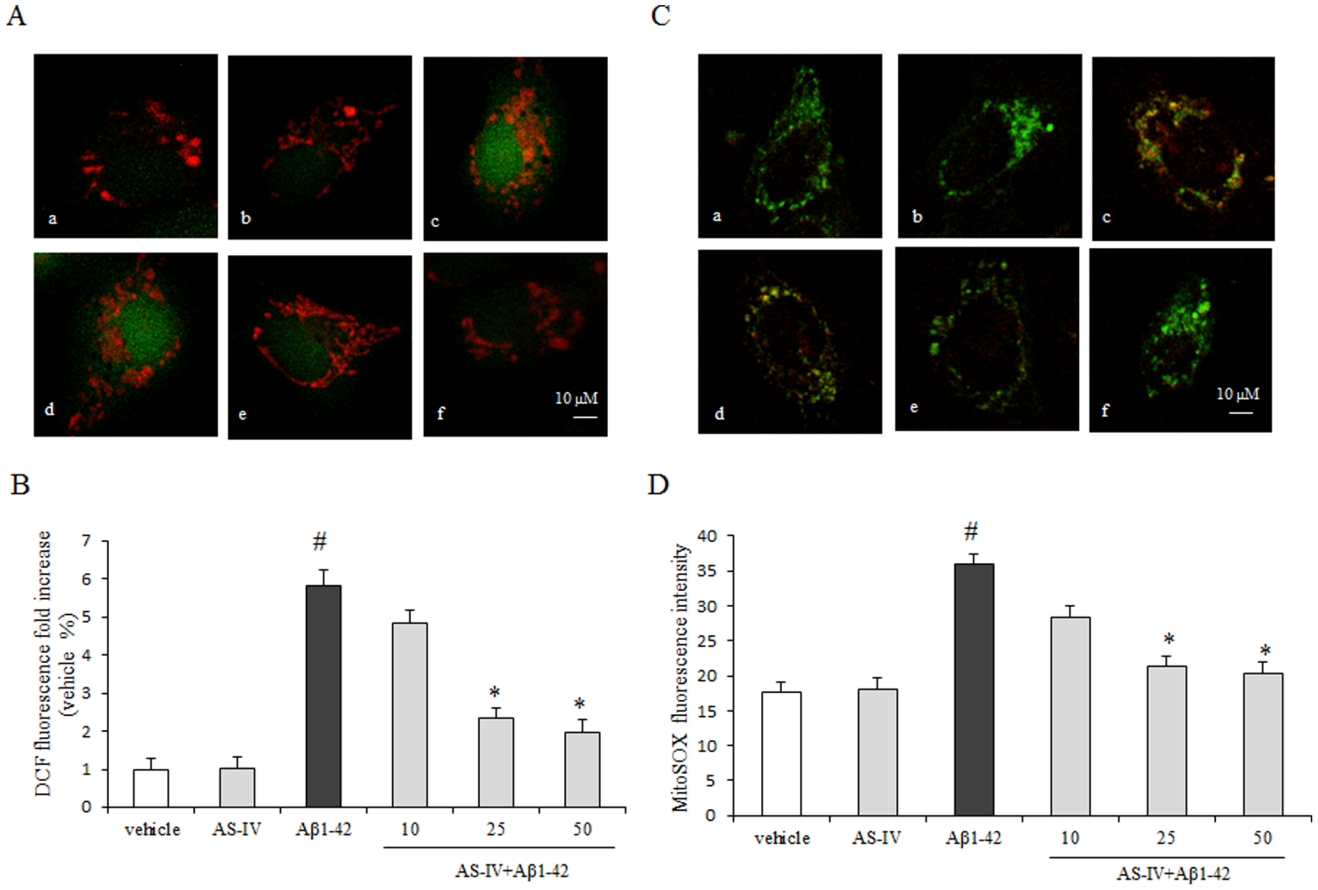
AS-IV attenuates Aβ1-42-induced increase of ROS generation in SK-N-SH cells. A. Confocal fluorescence images of intracellular ROS stained with DCFH-DA. Mitochondria were counterstained with Mitotracker Red (red fluorescence). (a) vehicle; (b) 50 µM AS-IV; (c) 5 µM Aβ1-42; (d, e, f) 10, 25, 50 µM AS-IV+ 5 µM Aβ1-42, respectively. B. Quantification analysis for DCFH-DA fluorescence intensity. ^#^ *P*<0.01 *vs* vehicle; ^*^ *P*<0.01 *vs* Aβ1-42 (n = 8). C. Confocal fluorescence images of mitochondrial superoxide stained with MitoSOX Red. Mitochondria were counterstained with Mitotracker Green (green fluorescence). (a) vehicle; (b) 50 µM AS-IV; (c) 5 µ µM Aβ1-42; (d, e, f) 10, 25, 50 µM AS-IV+ 5 µM Aβ1-42, respectively. D. Quantification analysis for MitoSOX Red fluorescence intensity ^#^ *P*<0.01 *vs* vehicle; ^*^ *P*<0.01 *vs* Aβ1-42 (n = 8). Scale bar = 10 µm.

To analyze whether AS-IV attenuates cell death by blocking ROS generation, we examined the level of intracellular ROS by using H_2_DCF-DA fluorescent dye. The lowest ROS level was detected from the vehicle group. Compared with the vehicle group, the Aβ1-42 group showed significantly higher level of green fluorescence intensity (*P*<0.01). In the presence of AS-IV, DCF intensity was significantly decreased in a dose-dependent manner compared with the Aβ1-42 group (*P*<0.01). However, pretreatment of AS-IV did not completely reverse the ROS level compared with that in the vehicle group (*P*<0.01) (Fig. 7A, B). 50 µM AS-IV alone treatment did not show an insult to SK-N-SH cells.

### Pretreatment with AS-IV decreased Aβ1-42-induced mitochondrial superoxide in SK-N-SH cells

To obtain evidence related to a reduction in the levels of mitochondrial superoxide, we stained live cells with MitoSOX Red fluorescent probe. Compared with the vehicle group, cells in the Aβ1-42 group demonstrated significant enhancement in red fluorescence signal (*P*<0.01). In the presence of AS-IV at 25 or 50 µM, MitoSOX Red fluorescence level was significantly reduced in a dose-dependent manner compared with that in the Aβ1-42 group (*P*<0.01) but the signal intensity was not back to the level of the vehicle group (*P*<0.01). There was no significant difference between the 10 µM AS-IV pretreatment group and the Aβ1-42 group (Fig. 7C, D). 50 µM AS-IV alone treatment did not show an insult to SK-N-SH cells.

### Bcl-2 and Bax are involved in AS-IV inhibiting the mPTP opening in the presence of Aβ1-42

**Figure 8:**
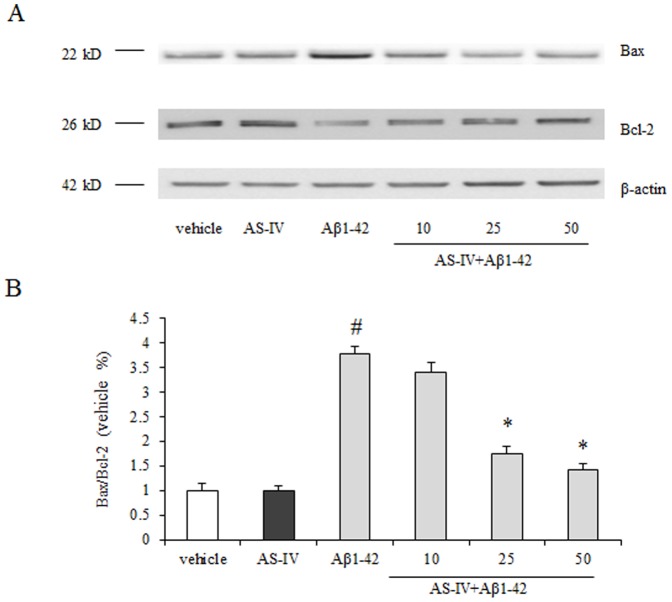
AS-IV inhibited Aβ1-42-induced increase of Bax/Bcl-2 ratio. (A) Western blot results of AS-IV on expression of Bax and Bcl-2. (B) The quantification of immunoreactive bands for Bax and Bcl-2 relative to β-actin and the Bax/Bcl-2 ratio was determined. ^#^ *P*<0.01 *vs* vehicle; ^*^ *P*<0.01 *vs* Aβ1-42 (n = 4).

The Bcl-2 family proteins are associated with apoptosis. In this study, we examined the expression of Bax and Bcl-2 by Western blot. Compared with the vehicle group, the Aβ1-42 group displayed lower expression of Bcl-2 and higher expression of Bax. The ratio of Bax/Bcl-2 in the Aβ1-42 group was significantly increased (*P*<0.01). Cells pretreated with AS-IV at 25 or 50 µM showed reversed ratios of Bax and Bcl-2 compared with cells in the Aβ1-42 group. The ratio of Bax/Bcl-2 in the AS-IV pretreated group was decreased compared with the Aβ1-42 group (*P*<0.01). No difference was observed between pretreatment of 10 µM AS-IV and Aβ1-42 treatment alone. 50 µM AS-IV alone treatment did not show an insult to SK-N-SH cells (Fig 8A, B).

## Discussion

As a main component of senior plaque, amyloid beta (Aβ) is an important hallmark of Alzheimer's disease. Previous studies have demonstrated Aβ could induce apoptotic and necrotic cell death [28]. In the present study, our data showed that SK-N-SH cells treated with 5 µM Aβ1-42 for 24 h displayed lower cell viability and higher apoptosis. Pretreatment of AS-IV at concentration of 25, 50 µM significantly decreased apoptotic cell numbers and improved cell viability in SK-N-SH cells in the presence of Aβ1-42. The results implicate that AS-IV plays a protective role in SK-N-SH cells resisting Aβ1-42 toxicity. Similarly, Zhang and colleagues report that AS-IV showed protective effect against MPP^+^-induced toxicity in their studies on PD model [29].

As the energy powerhouse of eukaryotic cells, the integrity of structure and healthy functions of mitochondria are essential to ensure cellular energy supplies and cell survival. Under pathological conditions, structural damage and subsequent dysfunction in mitochondria lead to irreversible death in eukaryotic cells [30]. During the process of mitochondria-mediated apoptosis, prior to other cellular alterations, mitochondria display earlier impairments such as a reduction in ATP production, loss of mitochondrial membrane potential (Δ*Ψ*m) and decreased activity of mitochondrial enzymes. A number of studies showed that dissipation of Δ*Ψ*m and mitochondrial structure damage appeared in Aβ-induced neurotoxicity [31], [32]. Here, our data showed that Aβ1-42 decreased Δ*Ψ*m, reduced ATP level and down-regulated of CcO activity which indicated that Aβ1-42 induced apoptosis in SK-N-SH cells through a mitochondrial pathway. These mitochondrial damages were significantly reversed by pretreatment of AS-IV at concentrations of 25 and 50 µM. Therefore, we hypothesized that the protection of AS-IV against Aβ1-42 in SK-N-SH cells owed to maintaining Δ*Ψ*m, CcO activity and ATP generation.

It is well known that cytochrome c is released from outer mitochondrial membrane, following the dissipation of Δ*Ψ*m. The dissipation of Δ*Ψ*m is an earlier event when neuronal cells are exposed to Aβ1-42 [33], [34]. In this study, the results showed that the expression of cytochrome c in cytosol was significantly increased in the Aβ group compared with those from the vehicle group. Pretreatment of AS-IV significantly prevented the cytochrome c release from mitochondria to cytosol compared with Aβ1-42 treatment alone. Several apoptosis-associated proteins, such as caspase-9 and caspase-3, are substrates for cytochrome c and could be cleaved by cytochrome c [35]. Our data showed Aβ1-42 treated cells displayed significantly increased levels of cleaved caspase-3 compared with cells in the vehicle group. As expected, pretreatment with AS-IV significantly reduced the levels of cleaved caspase-3 compared with that in the Aβ1-42 group. Taken together, these results indicate that AS-IV protects SK-N-SH cells against Aβ1-42 toxicity by reducing cytochrome c release and avoid apoptosis-associated proteins activation.

Besides supplying cellular energy, mitochondria are involved in a wide range of important cellular processes. Mitochondria exert an important physiological function in regulating intracellular Ca^2+^ homeostasis [36]. Mitochondrial dysfunction occurs in response to Ca^2+^ overload or ROS accumulation. A large body of evidence indicates that the mitochondrial permeability transition pore (mPTP) opening is a key event in Aβ-induced neurotoxicity [37]–[39]. Therefore we detected whether AS-IV could inhibit the mPTP opening during Aβ1-42 treatment in SK-N-SH cells. Our results demonstrate that the mPTP opening is enhanced by Aβ1-42 in SK-N-SH cells, which is coincident with a previous study [10]. Notably, pretreatment of AS-IV significantly inhibited the opening of the mPTP in Aβ1-42 rich milieu. These data suggest that AS-IV protects mitochondria via inhibiting Aβ1-42-induced mPTP opening, which ultimately protects SK-N-SH cells from Aβ1-42.

In most of neurodegenerative events such as Alzheimer's disease, the balance of oxidation-reduction in neurons is disturbed and excessive reactive oxygen species (ROS) generated following mitochondria superoxide accumulation [40]. Mitochondria play a pivotal role in maintaining the balance of oxidation-reduction. Superoxide is generated from mitochondrial respiratory chain complex I and complex III. Under physiologic conditions, mitochondrial superoxide can be converted gradually to H_2_O or be converted to hydrogen peroxide (H_2_O_2_) in the cytosol [41]. In this study, we found that mitochondrial superoxide and intracellular ROS in Aβ1-42 treated SK-N-SH cells were significantly increased compared with cells in the vehicle group. The excessive ROS will further exaggerate mitochondria damages, including the collapse of the Δ*Ψ*m, outer mitochondrial membrane rupture and inactivation of mitochondrial metabolic enzymes [42]. Inspiringly, in this study, we found that pretreatment of AS-IV reduced intracellular ROS and mitochondrial superoxide in SK-N-SH cells treated with Aβ1-42. As mentioned above, ROS is one inducer to trigger the mPTP opening. Therefore, we speculate the protective effect of AS- IV on SK-N-SH cells treated with Aβ1-42 interrupts the vicious cycle between the mPTP- mediated mitochondrial damages and cellular oxidative stress.

Bcl-2 family proteins are related to apoptosis. Under pro-apoptotic condition, Bax inserts into the outer mitochondrial membrane from cytosol. Bax can bind with ANT and/or VDAC proteins to induce the mPTP opening. On the contrary, Bcl-2 can prevent the mPTP opening by inhibiting the interaction between Bax and the mPTP components proteins [43]. In the present study, the results showed that the expression of Bax was increased in Aβ1-42 treated SK-N-SH cells compared with cells in the vehicle group, while the expression of Bcl-2 was significantly decreased. Both changes resulted increasing the ratio of Bax/Bcl-2 in Aβ1-42 treated SK-N-SH cells. Pretreatment of AS-IV significantly decreased the expression of Bax and increased the expression of Bcl-2 induced by Aβ1-42. The ratio of Bax/Bcl-2 was reversed in the pretreatment of AS-IV. These results indicate that pretreatment of AS-IV could inhibit the mPTP opening by reducing the expression of Bax and enhancing the expression of Bcl-2. In addition, the recovery ratio of Bax/Bcl-2 indicates that AS-IV inhibits Bax mediated mPTP opening. Moreover, studies showed that intracellular ROS could increase Bax and decrease Bcl-2 by regulating their phosphorylation and ubiquitination [44]. The possible mechanism of AS-IV inhibiting Aβ1-42-induced mPTP opening might be due to reducing intracellular ROS followed by alterations of the expression of Bax and Bcl-2.

In summary, our data demonstrated AS-IV enhanced cell viability and decreased accumulation of mitochondria superoxide and intracellular ROS in an Aβ1-42 rich environment. AS-IV improved mitochondrial function, maintained mitochondrial membrane potential and suppressed the release of cytochrome c and the caspase-3 activation in SK-N-SH cells treated with Aβ1-42. AS-IV inhibited the mPTP opening and reduced the ratio of Bax/Bcl-2 in SK-N-SH cells treated with Aβ1-42. These results suggest that AS-IV exerts protective effects on SK-N-SH cells against mitochondria-mediated apoptosis by inhibiting the mPTP opening and regulating the expression of Bcl-2 family proteins in the presence of Aβ1-42. Our data provide evidence to support the protective effects of AS-IV on neuronal cells in neurodegeneration diseases. These results provide novel insights of AS-IV for the prevention and treatment of neurodegenerative disorders such as AD.

## References

[1] Goedert M, Spillantini MG (2006) A century of Alzheimer's disease. Science 314: 777–781 10.1126/science.1132814 17082447

[2] Kadowaki H, Nishitoh H, Urano F, Sadamitsu C, Matsuzawa A, et al (2005) Amyloid beta induces neuronal cell death through ROS-mediated ASK1 activation. Cell Death Differ 12: 19–24 10.1038/sj.cdd.4401528 15592360

[3] Takuma K, Yao J, Huang J, Xu H, Chen X, et al (2005) ABAD enhances Abeta-induced cell stress via mitochondrial dysfunction. FASEB J 19: 597–598 10.1096/fj.04-2582fje 15665036

[4] Wang-Dietrich L, Funke SA, Kuhbach K, Wang K, Besmehn A, et al (2013) The amyloid-beta oligomer count in cerebrospinal fluid is a biomarker for Alzheimer's disease. J Alzheimers Dis 34: 985–994 10.3233/JAD-122047 23313925

[5] Um JW, Nygaard HB, Heiss JK, Kostylev MA, Stagi M, et al (2012) Alzheimer amyloid-beta oligomer bound to postsynaptic prion protein activates Fyn to impair neurons. Nat Neurosci 15: 1227–1235 10.1038/nn.3178 22820466 PMC3431439

[6] Horiuchi M, Maezawa I, Itoh A, Wakayama K, Jin LW, et al (2012) Amyloid beta1-42 oligomer inhibits myelin sheet formation in vitro. Neurobiol Aging 33: 499–509 10.1016/j.neurobiolaging.2010.05.007 20594620 PMC3013291

[7] Chafekar SM, Hoozemans JJ, Zwart R, Baas F, Scheper W (2007) Abeta 1-42 induces mild endoplasmic reticulum stress in an aggregation state-dependent manner. Antioxid Redox Signal 9: 2245–2254 10.1089/ars.2007.1797 17979527

[8] Halestrap AP (2006) Calcium, mitochondria and reperfusion injury: a pore way to die. Biochem Soc Trans 34: 232–237 10.1042/BST20060232 16545083

[9] Baines CP, Kaiser RA, Purcell NH, Blair NS, Osinska H, et al (2005) Loss of cyclophilin D reveals a critical role for mitochondrial permeability transition in cell death. Nature 434: 658–662 10.1038/nature03434 15800627

[10] Qu M, Zhou Z, Chen C, Li M, Pei L, et al (2012) Inhibition of mitochondrial permeability transition pore opening is involved in the protective effects of mortalin overexpression against beta-amyloid-induced apoptosis in SH-SY5Y cells. Neurosci Res 72: 94–102 10.1016/j.neures.2011.09.009 22001761

[11] Ren R, Zhang Y, Li B, Wu Y (2011) Effect of beta-amyloid (25-35) on mitochondrial function and expression of mitochondrial permeability transition pore proteins in rat hippocampal neurons. J Cell Biochem 112: 1450–1457 10.1002/jcb.23062 21321998

[12] Du H, Guo L, Fang F, Chen D, Sosunov AA, et al (2008) Cyclophilin D deficiency attenuates mitochondrial and neuronal perturbation and ameliorates learning and memory in Alzheimer's disease. Nat Med 14: 1097–1105 10.1038/nm.1868 18806802 PMC2789841

[13] Zamzami N, Brenner C, Marzo I, Susin SA, Kroemer G (1998) Subcellular and submitochondrial mode of action of Bcl-2-like oncoproteins. Oncogene 16: 2265–2282 10.1038/sj.onc.1201989 9619836

[14] Brustovetsky T, Li T, Yang Y, Zhang JT, Antonsson B, et al (2010) BAX insertion, oligomerization, and outer membrane permeabilization in brain mitochondria: role of permeability transition and SH-redox regulation. Biochim Biophys Acta 1797: 1795–1806 10.1016/j.bbabio.2010.07.006 20655869 PMC2933961

[15] Chen Q, Lesnefsky EJ (2011) Blockade of electron transport during ischemia preserves bcl-2 and inhibits opening of the mitochondrial permeability transition pore. FEBS Lett 585: 921–926 10.1016/j.febslet.2011.02.029 21354418 PMC3076511

[16] Kawamata J, Shimohama S (2011) Stimulating nicotinic receptors trigger multiple pathways attenuating cytotoxicity in models of Alzheimer's and Parkinson's diseases. J Alzheimers Dis 24 Suppl 295–109 10.3233/JAD-2011-110173 21403387

[17] Liu T, Jin H, Sun QR, Xu JH, Hu HT (2010) Neuroprotective effects of emodin in rat cortical neurons against beta-amyloid-induced neurotoxicity. Brain Res 1347: 149–160 10.1016/j.brainres.2010.05.079 20573598

[18] Guo L, Du H, Yan S, Wu X, McKhann GM, et al (2013) Cyclophilin D deficiency rescues axonal mitochondrial transport in Alzheimer's neurons. PLoS One 8: e54914 10.1371/journal.pone.0054914 23382999 PMC3561411

[19] Nam JH, Park KW, Park ES, Lee YB, Lee HG, et al (2012) Interleukin-13/-4-induced oxidative stress contributes to death of hippocampal neurons in abeta1-42-treated hippocampus in vivo. Antioxid Redox Signal 16: 1369–1383 10.1089/ars.2011.4175 22248368

[20] Zhu X, Chen C, Ye D, Guan D, Ye L, et al (2012) Diammonium glycyrrhizinate upregulates PGC-1alpha and protects against Abeta1-42-induced neurotoxicity. PLoS One 7: e35823 10.1371/journal.pone.0035823 22540007 PMC3335163

[21] Polat E, Bedir E, Perrone A, Piacente S, Alankus-Caliskan O (2010) Triterpenoid saponins from Astragalus wiedemannianus Fischer. Phytochemistry 71: 658–662 10.1016/j.phytochem.2009.11.013 20060986

[22] Zhang N, Wang XH, Mao SL, Zhao F (2011) Astragaloside IV improves metabolic syndrome and endothelium dysfunction in fructose-fed rats. Molecules 16: 3896–3907 10.3390/molecules16053896 21555978 PMC6263341

[23] Zhang DW, Bian ZP, Xu JD, Wu HF, Gu CR, et al (2012) Astragaloside IV alleviates hypoxia/reoxygenation-induced neonatal rat cardiomyocyte injury via the protein kinase a pathway. Pharmacology 90: 95–101 10.1159/000339476 22797566

[24] Chan WS, Durairajan SS, Lu JH, Wang Y, Xie LX, et al (2009) Neuroprotective effects of Astragaloside IV in 6-hydroxydopamine-treated primary nigral cell culture. Neurochem Int 55: 414–422 10.1016/j.neuint.2009.04.012 19409437

[25] Ren S, Zhang H, Mu Y, Sun M, Liu P (2013) Pharmacological effects of Astragaloside IV: a literature review. J Tradit Chin Med 33: 413–416. 24024343 10.1016/s0254-6272(13)60189-2

[26] Cheng CY, Yao CH, Liu BS, Liu CJ, Chen GW, et al (2006) The role of astragaloside in regeneration of the peripheral nerve system. J Biomed Mater Res A 76: 463–469 10.1002/jbm.a.30249 16315188

[27] Stine WB Jr, Dahlgren KN, Krafft GA, LaDu MJ (2003) In vitro characterization of conditions for amyloid-beta peptide oligomerization and fibrillogenesis. J Biol Chem 278: 11612–11622 10.1074/jbc.M210207200 12499373

[28] Dartigues JF (2009) Alzheimer's disease: a global challenge for the 21st century. Lancet Neurol 8: 1082–1083 10.1016/S1474-4422(09)70298-4 19909903

[29] Zhang ZG, Wu L, Wang JL, Yang JD, Zhang J, et al (2012) Astragaloside IV prevents MPP(+)-induced SH-SY5Y cell death via the inhibition of Bax-mediated pathways and ROS production. Mol Cell Biochem 364: 209–216 10.1007/s11010-011-1219-1 22278385

[30] Baines CP (2010) Role of the mitochondrion in programmed necrosis. Front Physiol 1: 156 10.3389/fphys.2010.00156 21423395 PMC3059973

[31] Du H, Guo L, Yan S, Sosunov AA, McKhann GM, et al (2010) Early deficits in synaptic mitochondria in an Alzheimer's disease mouse model. Proc Natl Acad Sci U S A 107: 18670–18675 10.1073/pnas.1006586107 20937894 PMC2972922

[32] Martin LJ (2012) Biology of mitochondria in neurodegenerative diseases. Prog Mol Biol Transl Sci 107: 355–415 10.1016/B978-0-12-385883-2.00005-9 22482456 PMC3530202

[33] Fernandez-Morales JC, Arranz-Tagarro JA, Calvo-Gallardo E, Maroto M, Padin JF, et al (2012) Stabilizers of neuronal and mitochondrial calcium cycling as a strategy for developing a medicine for Alzheimer's disease. ACS Chem Neurosci 3: 873–883 10.1021/cn3001069 23173068 PMC3503342

[34] Krstic D, Knuesel I (2013) Deciphering the mechanism underlying late-onset Alzheimer disease. Nat Rev Neurol 9: 25–34 10.1038/nrneurol.2012.236 23183882

[35] Garrido C, Galluzzi L, Brunet M, Puig PE, Didelot C, et al (2006) Mechanisms of cytochrome c release from mitochondria. Cell Death Differ 13: 1423–1433 10.1038/sj.cdd.4401950 16676004

[36] Halestrap AP, Pasdois P (2009) The role of the mitochondrial permeability transition pore in heart disease. Biochim Biophys Acta 1787: 1402–1415 10.1016/j.bbabio.2008.12.017 19168026

[37] Du H, Guo L, Zhang W, Rydzewska M, Yan S (2011) Cyclophilin D deficiency improves mitochondrial function and learning/memory in aging Alzheimer disease mouse model. Neurobiol Aging 32: 398–406 10.1016/j.neurobiolaging.2009.03.003 19362755 PMC3304024

[38] Bartley MG, Marquardt K, Kirchhof D, Wilkins HM, Patterson D, et al (2012) Overexpression of amyloid-beta protein precursor induces mitochondrial oxidative stress and activates the intrinsic apoptotic cascade. J Alzheimers Dis 28: 855–868 10.3233/JAD-2011-111172 22133762 PMC4679200

[39] Du H, Yan SS (2010) Mitochondrial permeability transition pore in Alzheimer's disease: cyclophilin D and amyloid beta. Biochim Biophys Acta 1802: 198–204 10.1016/j.bbadis.2009.07.005 19616093 PMC3280723

[40] Ebenezer PJ, Weidner AM, LeVine H 3rd, Markesbery WR, Murphy MP, et al (2010) Neuron specific toxicity of oligomeric amyloid-beta: role for JUN-kinase and oxidative stress. J Alzheimers Dis 22: 839–848 10.3233/JAD-2010-101161 20858948 PMC3412400

[41] Sena LA, Chandel NS (2012) Physiological roles of mitochondrial reactive oxygen species. Mol Cell 48: 158–167 10.1016/j.molcel.2012.09.025 23102266 PMC3484374

[42] Federico A, Cardaioli E, Da Pozzo P, Formichi P, Gallus GN, et al (2012) Mitochondria, oxidative stress and neurodegeneration. J Neurol Sci 322: 254–262 10.1016/j.jns.2012.05.030 22669122

[43] Kroemer G, Galluzzi L, Brenner C (2007) Mitochondrial membrane permeabilization in cell death. Physiol Rev 87: 99–163 10.1152/physrev.00013.2006 17237344

[44] Li D, Ueta E, Kimura T, Yamamoto T, Osaki T (2004) Reactive oxygen species (ROS) control the expression of Bcl-2 family proteins by regulating their phosphorylation and ubiquitination. Cancer Sci 95: 644–650. 15298726 10.1111/j.1349-7006.2004.tb03323.x PMC11158795

